# Blood transfusion and high-order cesarean delivery; Report from a developing country

**DOI:** 10.12669/pjms.35.6.539

**Published:** 2019

**Authors:** Shahida Abbas, Saba Mughal, Syeda Namayah Fatima Hussain, Nazli Hossain

**Affiliations:** 1 Shahida Abbas, FCPS, MCPS. Department of Obstetrics & Gynecology, Holy Family Hospital, Karachi, Pakistan; 2 Saba Mughal, Department of Research, DOW University of Health Sciences, Karachi, Pakistan; 3 Syeda Namayah Fatima Hussain, 4^th^ Year, MBBS. Liaquat National Hospital & Medical College, Karachi, Pakistan; 4 Nazli Hossain, FCPS, MBE. Department of Obstetrics & Gynecology, DOW University of Health Sciences, Karachi, Pakistan

**Keywords:** Blood transfusion, Cesarean section, Risk factors

## Abstract

**Background and Objective:**

Blood loss in cesarean deliveries has already been established in previous researches but a detailed insight into the correlates has not been done. This study examined whether the number of previous Cesarean sections is related to the need for blood transfusion, and risk factors for blood transfusion.

**Methods:**

A retrospective review of 239 females who had undergone two or more Cesarean sections during the time period of 2015-2018 was done. Data collected included type of surgery (elective or emergency), age, parity, body mass index, estimated blood loss, operating time, level of surgeon, presence or absence of adhesions and number of transfused packed cell volume.

**Results:**

About 9.2% patients received blood transfusion with an estimated average blood loss of 618.18 ml. Patients with adhesions from previous surgery, presence of placenta previa, multiparity were significantly likely to receive blood transfusion. It was found that women with more than two caesarian sections had high proportion of blood transfusion as compared to women who had two caesarian sections. However non-significant difference was observed in numbers of caesarean sections with blood transfusion.

**Conclusion:**

Women undergoing Cesarean sections combined with any of the risk factors like increased body mass index, dense adhesions, uterine atony, hypertension and presence of placenta previa, were found to be at increased risk for a need for blood transfusions.

## INTRODUCTION

Maternal mortality, maternal morbidity and maternal near miss have been linked to hemorrhage as their leading cause in developing nations.^1^ Globally the Cesarean section rate has increased, with the highest rates quoted for developed world.^2^ Pakistan has also seen a sharp rise in rate of Cesarean section. More than 80% of obstetric hemorrhages occur postpartum (PPH) and are responsible for 25% of maternal deaths each year.^3^ Cesarean section is also identified as an important cause of PPH, carrying risk for major intraoperative blood loss.^4^ In obstetric practice, this makes cesarean section an important indicator for blood transfusion.

As the number of cesarean sections CS increase, the complexity and complications also increase simultaneously, and so does the need for blood transfusions. Other factors might also significantly contribute to blood loss during cesarean sections CS such as maternal age, body mass index (BMI), comorbidities like fibroid uterus, experience of surgeon, and duration of surgery. The risk of transfusion increases as the number of cesarean section*s* increases. Abdelazim I et al., found that risk for blood transfusion increased 4.7%, in women having more than three Cesarean sections.^4^ However, preparing cross-matched blood in anticipation not only causes an economic burden but also scarcity of blood especially in a developing country like Pakistan. Studies have shown wastage and over ordering of blood products leading to a loss of not just money but also manpower and time of blood bank resources. Subsequently, this may lead to deprivation of blood to people in time of need since the country lacks a reliable donor base as well as a regulatory and governance setup. Also it adds to the emotional stress of the attendants who brought the patient. Due to paucity of data in our region, it becomes essential to evaluate the effectiveness of arranging blood, and blood products in cases of most common obstetrical surgical procedure of Cesarean section.

This study was aimed at identifying risk factors for blood transfusion in women undergoing repeat cesarean section. High order cesarean section was defined as women having 2 or more than two previous operative deliveries.

## METHODS

This retrospective study was carried out at a secondary care center. The study period was from January 2015 to December 2018. The hospital has a well established medical record system. The hospital receives patients mainly from the city. The number of annual deliveries of the hospital are 1500. The delivery room is managed by midwives and registrars. The operative delivery is carried out by the consultant, with at least a minimum of five years’ experience. The hospital provides emergency services for twenty-four hours. Women having two or more cesarean sections were included in the study. Medical records were evaluated for age, parity, body mass index, duration of surgery, emergency or elective procedure, number of transfusions of blood and blood products, presence or absence of adhesions, and presence or absence of placenta previa. Also included were details of newborn including weight and APGAR score. Women were divided in two groups on the basis of whether they received blood transfusion or not. The study was approved by the Hospital’s ethics committee. (Ethics Committee approval June 10, 2017).

### Statistical analysis

The statistical analysis was carried out using Statistical Package for Social Sciences (SPSS) version 16.0. Frequency and percentage were used to describe categorical variables such as parity, number of previous Cesarean Section*s* (LSCS), type of surgery, hypertension, diabetes mellitus, adhesions, uterine atony and placenta previa. Range, mean and standard deviation (SD) were reported to describe continuous variables such as age, gestational age, Body Mass Index (BMI), estimated blood loss, operative time, hospital stay, birth weight, APGAR score at 1-minute and APGAR score at 5-minute. Normality of continuous variables was checked using Shapiro-Wilk test and Mann-Whitney U test was applied to check mean differences between groups of blood transfusion. Chi-square test was run to check association between categorical variables and blood transfusion. Univariate and multivariate binary logistic regression analyses were carried out, unadjusted and adjusted odds ratios (OR) with 95% confidence intervals (CI) were reported for the blood transfusion by other independent factors. Multivariate model was adjusted for all those covariates whose p-values were found to be less than 0.25 in univariate logistic model. All test results having p-values less than or equal to 0.05 level of significance were considered statistically significant.

## RESULTS

A total of 239 pregnant females were included in this study who underwent two or more cesarean sections. Total number of Cesarean sections during the study period was 2482. Average age of females was 30.13 years with SD ±4.08 years and ranged between 21 and 43 years whereas average BMI of females was 28.29 with SD ±5.71 and ranged between 18.1 and 59.5 Overall, 9.2% (n=22) patients received a blood transfusion. Patients who underwent their third, fourth and fifth cesarean section were 76.6% (n=183), 20.9% (n=50) and 2.5%, (n=6), respectively. Majority of study participants 85.4% (n=204) had elective surgery. Mild, moderate and dense adhesions were observed in 154 (64.4%), 18 (7.5%), and 67 (28%) women respectively. Patients who reported no/mild, moderate and dense adhesions were 64.4% (n=154), 7.5% (n=18) and 28.0% (n=67), respectively (Table-I).

**Table I:** Baseline characteristics of the participants (n=239).

Characteristics	n	%
***Blood transfusion***		
No	217	90.8
Yes	22	9.2
***Parity***		
2	158	66.1
3	58	24.3
4	23	9.6
***No. of previous LSCS***		
2	183	76.6
>2	56	23.4
***Type of surgery***		
Elective	204	85.4
Emergency	35	14.6
***Hypertension***		
No	215	90.0
Yes	24	10.0
***Diabetes mellitus***		
No	217	90.8
Yes	22	9.2
***Adhesions***		
No/mild	154	64.4
Moderate	18	7.5
Dense	67	28.0
***Uterine Atony***		
No	232	97.1
Yes	7	2.9
***Placenta previa***		
No	236	98.7
Yes	3	1.3

BMI: Body Mass Index, LSCS: Lower Segment Cesarean Section.

The average estimated blood loss was 618.18 ml with SD ±895.29 in transfused group and 248.43 ml with SD ±132.42 in non-transfused group. The average blood loss was significantly different between the groups (Mean difference=369.75). BMI (Mean difference=1.99) and hospital stay in days (Mean difference=0.71) also showed statistically significant mean differences between the groups of blood transfusion. Results showed that women with more than two caesarian sections had high proportion of blood transfusion as compared to women who had two caesarian sections. However non-significant difference observed in number of caesarean sections with blood transfusion. It was observed that those patients who had dense adhesions were transfused more compared to those who had no/mild adhesions. Whereas, it was found that adhesions were statistically significantly associated with blood transfusion (χ^2^=15.24). The presence of placenta previa (χ^2^=12.00), parity (χ^2^=8.70) and uterine atony (χ^2^=19.83) were also significantly associated with blood transfusion (Table-II).

**Table II:** Demographic characteristics of females undergoing cesarean sections by blood transfusion (n=239).

Variables	Transfused (n = 22)	Not transfused (n = 217)	p-value*
Age in years (Mean ± SD)	30.55 ± 4.03	30.09 ± 4.10	0.753
Gestational age in weeks (Mean ± SD)	36.59 ± 0.85	36.63 ± 2.48	0.272
BMI (Mean ± SD)	30.10 ± 4.51	28.11 ± 5.80	0.020
Estimated blood loss in ml (Mean ± SD)	618.18 ± 895.29	248.43 ± 132.42	< 0.001
Operative time in minutes (Mean ± SD)	61.59 ± 35.77	46.49 ± 7.42	0.128
Hospital stay in days (Mean ± SD)	3.95 ± 1.29	3.24 ± 0.62	0.005
***Parity, n (%)***			
2	12 (7.6)	146 (92.4)	0.013^†^
3	4 (6.9)	54 (93.1)	
4	6 (26.1)	17 (73.9)	
***No. of previous LSCS, n (%)***			
2	15 (8.2)	168 (91.8)	0.330^†^
>2	7 (12.5)	49 (87.5)	
***Type of surgery, n (%)***			
Elective	17 (8.3)	187 (91.7)	0.337^‡^
Emergency	5 (14.3)	30 (85.7)	
***Hypertension, n (%)***			
No	17 (7.9)	198 (92.1)	0.054^‡^
Yes	5 (20.8)	19 (79.2)	
***Diabetes mellitus, n (%)***			
No	21 (9.7)	196 (90.3)	0.703^‡^
Yes	1 (4.5)	21 (95.5)	
***Adhesions, n (%)***			
No/mild	7 (4.5)	147 (95.5)	< 0.001**
Moderate	1 (5.6)	17 (94.4)	
Dense	14 (20.9)	53 (79.1)	
***Uterine Atony, n (%)***			
No	18 (7.8)	214 (92.2)	0.002^‡^
Yes	4 (57.1)	3 (42.9)	
***Placenta previa, n (%)***			
No	20 (8.5)	216 (91.5)	0.023^‡^
Yes	2 (66.7)	1 (33.3)	

* p-value has been calculated using Mann-Whitney U test,

† p-value has been calculated using Chi-square test,

‡ p-value has been calculated using Fisher’s exact test.

The average birth weight of infants was slightly higher in transfused group as compared to not transfused group. However, birth weight, 1-minute and 5-minute Apgar scores did not show any statistically significant mean differences between the groups of blood transfusion (Table-III).

**Table III:** Infant characteristics in females undergoing cesarean section (n=239).

Characteristics	Transfused (n = 22)	Not transfused (n = 217)	p-value§
Birth weight in kg (Mean ± SD)	2.84 ± 0.36	2.76 ± 0.41	0.449
Apgar score at 1-min (Mean ± SD)	7.91 ± 0.29	7.79 ± 0.67	0.398
Apgar score at 5-min (Mean± SD)	8.86 ± 0.46	8.73 ± 0.77	0.512

§p-value has been calculated using Mann-Whitney U test.

Univariate analyses revealed that patients with higher parity (parity = 4) were more likely to receive a blood transfusion as compared to those who had lower parity (parity = 2) and those patients who had dense adhesions were more likely to be transfused as compared to those who had no/mild adhesions. It was also found that operative time, hospital stay, hypertension, uterine atony and placenta previa had statistically significant association with blood transfusion. After adjusting the multivariate logistic regression mode, it was again observed that hypertensive females were more likely to receive a blood transfusion than non-hypertensive females and females who had dense adhesions were more prone to be transfused as compared to those females who had no/mild adhesions. However, uterine atony and placenta previa remained consistent and showed significant association with higher risk for blood transfusion (Table-IV).

**Table IV:** Odds ratio for blood transfusion by important risk factors (n=239).

Characteristics	Blood transfusion			

	ORa (95% CI)	p value	ORb (95% CI)	p-value
Age in years	1.03 (0.92 - 1.14)	0.616	-	
Gestational age in weeks	0.99 (0.84 - 1.18)	0.946	-	
BMI	1.05 (0.99 - 1.13)	0.123	1.04 (0.93 - 1.15)	0.532
Estimated blood loss in ml	1.00 (1.00 - 1.01)	< 0.001	1.00 (1.00 - 1.01)	0.089
Operative time in minutes	1.07 (1.02 - 1.12)	0.008	1.06 (0.99 - 1.13)	0.084
Hospital stay in days	2.68 (1.59 - 4.52)	< 0.001	1.59 (0.77 - 3.29)	0.207
***Parity***				
2	Ref		Ref	
3	0.90 (0.28 - 2.92)	0.862	0.26 (0.04 - 1.64)	0.152
4	4.29 (1.43 - 12.92)	0.009	0.71 (0.16 - 3.18)	0.653
***No. of previous LSCS***				
2	Ref			
>2	1.60 (0.62 - 4.14)	0.333	-	
***Type of surgery***				
Elective	Ref			
Emergency	1.83 (0.63 - 5.34)	0.266	-	
***Hypertension***				
No	Ref		Ref	
Yes	3.06 (1.02 - 9.23)	0.046	6.31 (1.45 - 27.53)	0.014
***Diabetes mellitus***				
No	Ref			
Yes	0.44 (0.06 - 3.47)	0.439	-	
***Adhesions***				
No/mild	Ref		Ref	
Moderate	1.24 (0.14 - 10.65)	0.848	6.45 (0.48 - 86.38)	0.159
Dense	5.55 (2.12 - 14.48)	< 0.001	13.62 (2.63 - 70.66)	0.002
***Uterine Atony***				
No	Ref		Ref	
Yes	15.85 (3.29 - 76.37)	0.001	49.03 (4.16 - 578.30)	0.002
***Placenta previa***				
No	Ref		Ref	
Yes	21.60 (1.87 - 248.75)	0.014	77.25 (1.72 - 3464.24)	0.025

ORa: Unadjusted odds ratio, ORb: Odds ratio adjusted for BMI, estimated blood loss, operative time, hospital stay, parity, hypertension, adhesions, uterine atony and placenta previa, CI: confidence interval.

## DISCUSSION

Cesarean section is associated with increased morbidity and mortality. Common complications include increased blood loss, sepsis, ileus, rupture of previous uterine scar and iatrogenic injuries to bladder and bowel. As a precautionary measure, the usual protocol in the hospital is to arrange minimum of one unit of packed red cell prior to surgery. The frequency of blood transfusion in this study was found to be 9.2%. A study from neighboring country India quoted a higher rate of 12%.^5^

We found an increased risk for transfusion in women with increased body mass index, though it was not statistically significant. Paglia MJ et al. found an increased prevalence of PPH in cohort of more than 12,000 women, who had BMI <30.^6^ Women with increased parity were also found to have more likelihood of receiving blood transfusion. This may be attributed to increased risk of uterine atony in this group of women. Uterine atony leading to severe obstetric hemorrhage has also been observed in a large birth registry cohort from Norway.^7^ The risk for transfusion increases in women with placenta previa. This has also been seen by other investigators.^8^,^9^ Other risk factors which have been identified for increased risk of blood transfusion includes general anesthesia, emergency cesarean section, use of anticoagulants during antepartum period and eclampsia. Majority of CD in our study were elective surgeries. We did observe an increased risk of transfusion in women with hypertension.

The risk of transfusion was found to be increased in the presence of adhesions. It was more increased with dense adhesions, also resulting in an increase in the procedure duration. Both the factors significantly increased the risk for blood transfusion. Adhesions are common after pelvic surgery. It has been found that *the* risk increased from 46% in women with previous one CS, to 83% in women with previous four CS.^10^ Presence of adhesions after CS, depends upon a number of factors including technique, expertise of surgeon, infection after procedure, duration of procedure. Lyell DJ, found a decreased prevalence of adhesions in women who had closure of peritoneum at the time of CS.^11^ Peritoneal closure was protective against both mild (three fold) and dense adhesions (five fold). In our study, the CS were carried out by more than six different consultants, with no uniform policy on peritoneal closure, hence we can not comment upon the fact that whether peritoneal closure at the time of CS is associated with decreased risk of adhesions at the time of CD. Moreover there is still no consensus on suturing or not suturing visceral and parietal peritoneum, and the type of suture material which may reduce adhesions.^12^

We had only two patients who had placenta previa with repeat CS. It is generally accepted that women with placenta previa do have an increased risk of transfusion, more so in the presence of adherent placenta. The majority of women who received transfusion in the study had adhesions, uterine atony and hypertension in the study.

We had a sizeable number of women who underwent repeat CS. The rate of transfusion comes around 9.2%. This appears higher than other reported studies. The study does contradict the old theory that women with previous cesarean scar require blood transfusion. The risk of transfusion has been found to be increased in women undergoing repeat CD, after five Cesarean deliveries.^13^ The risk factors identified from our study include parity, BMI, uterine atony, hypertension and presence of placenta previa.

In a large cohort of more than 50,000 women investigators combined the antepartum and intrapartum risk factors for peripartum transfusion.^14^ Anemia, abruption, general anesthesia and abnormal placentation (OR 92, CI 57.4- 147.6) were identified as the risk factors in this combined model.

Though our data is limited, and is retrospective in nature, it does identify the main indications for blood transfusion in women undergoing high order CS. It also provides a guideline for the caregivers when to order blood arrangement in women undergoing repeat CS.

### Authors’ Contribution:

**SA and NH** designed the study.

**SNFH** was responsible for data entry and initial write up.

**SM** conducted the statistical analysis.

**NH** Prepared the final version of manuscript and is responsible for integrity of research.
